# Prospective Memory, Personality, and Individual Differences

**DOI:** 10.3389/fpsyg.2013.00130

**Published:** 2013-03-22

**Authors:** Bob Uttl, Carmela A. White, Daniela Wong Gonzalez, Joanna McDouall, Carrie A. Leonard

**Affiliations:** ^1^Department of Psychology, Mount Royal UniversityCalgary, AB, Canada

**Keywords:** prospective memory, big five, personality, individual differences, meta-analysis, verbal intelligence, retrospective memory

## Abstract

A number of studies investigating the relationship between personality and prospective memory (ProM) have appeared during the last decade. However, a review of these studies reveals little consistency in their findings and conclusions. To clarify the relationship between ProM and personality, we conducted two studies: a meta-analysis of prior research investigating the relationships between ProM and personality, and a study with 378 participants examining the relationships between ProM, personality, verbal intelligence, and retrospective memory. Our review of prior research revealed great variability in the measures used to assess ProM, and in the methodological quality of prior research; these two factors may partially explain inconsistent findings in the literature. Overall, the meta-analysis revealed very weak correlations (*r*s ranging from 0.09 to 0.10) between ProM and three of the Big Five factors: Openness, Conscientiousness, and Agreeableness. Our experimental study showed that ProM performance was related to individual differences such as verbal intelligence as well as to personality factors and that the relationship between ProM and personality factors depends on the ProM subdomain. In combination, the two studies suggest that ProM performance is relatively weakly related to personality factors and more strongly related to individual differences in cognitive factors.

## Introduction

Prospective memory (ProM) enables us to make plans, to retain them, and to bring them back into consciousness at the right time and place (Brandimonte et al., 2001; Graf and Uttl, 2001; Uttl, 2008). We use ProM for many different everyday tasks such as buying groceries en route home, watching a bathtub so it does not overflow, and picking up a child from daycare. ProM is divided into several subdomains: ProM proper or episodic ProM, vigilance/monitoring, and habitual ProM (Harris, 1984; Brandimonte et al., 2001; Graf and Uttl, 2001; Uttl, 2008). Episodic ProM brings a previously made plan back to consciousness in the appropriate context (e.g., upon noticing a supermarket) or at the appropriate time; it allows us to recognize ProM cues as signs of the previously formed plan (Graf and Uttl, 2001). In contrast, vigilance/monitoring requires that the plan remain in consciousness during the retention period. Habitual ProM is similar to episodic ProM but the plan must be executed repeatedly; each time it must leave consciousness and be brought back by an appropriate context/ProM cue or time. Accordingly, buying groceries en route home requires episodic ProM, watching a bathtub so it does not overflow requires vigilance/monitoring, and picking up a child from daycare every day requires habitual ProM.

Interest in how individual differences influence ProM performance has been in the forefront of ProM research at least since Einstein and McDaniel’s (1990) provocative claim that ProM is an “exciting exception to typically found age-related decrements in memory” (p. 724). Although ProM has since been found to decline with aging at least as much as retrospective memory, if not more (Uttl, 2008, 2011), Einstein and McDaniel’s claim sparked widespread interest in individual differences – age – and performance on ProM tasks. Moreover, a number of researchers reported correlations between intelligence and performance on ProM tasks (Maylor, 1996; Cherry and LeCompte, 1999; Uttl, 2006; Uttl and Kibreab, 2011). Associations between ProM and intelligence were strong enough to partially explain the lack of age declines found in a number of studies that confounded age with intelligence, and compared the performance of highly intelligent older adults with not so intelligent younger adults (Uttl, 2008, 2011). Similarly, researchers have begun to investigate associations between ProM and other cognitive factors, such as processing resources (e.g., Uttl, 2006) and working memory (Smith, 2003; Smith and Bayen, 2005). Most recently, they have become interested in the associations between non-cognitive factors such as personality and lifestyle and ProM (Searleman, 1996; Heffernan and Ling, 2001; Salthouse et al., 2004; Pearman and Storandt, 2005; Cuttler and Graf, 2007; Smith et al., 2011; Uttl and Kibreab, 2011).

In an introduction to one of the first studies of personality and ProM, Searleman (1996) wrote: “people vary tremendously in their ability to successfully carry out many types of ProM tasks – some are astonishingly proficient at such tasks, whereas others are absolutely terrible at them” (p. 112). In turn, he investigated the relationship between four personality factors – the Type A/B personality, obsessiveness-compulsiveness, self-actualization, and self-monitoring behavior – that he believed might logically be related to performance on ProM tasks. However, Searleman found that only the Type A/B factor was weakly related to ProM performance, with the Type A individuals outperforming the Type B individuals on an interpersonal task (e.g., reminding an experimenter to make a phone call) but not on a non-interpersonal task (e.g., leaving a message on the answering machine 1 week later).

A number of studies investigating the associations between ProM and personality followed with seemingly inconsistent results (Heffernan and Ling, 2001; Salthouse et al., 2004; Pearman and Storandt, 2005; Cuttler and Graf, 2007; Arana et al., 2008; Smith et al., 2011; Uttl and Kibreab, 2011). To illustrate, discussing the results of prior studies as well as their own study, Smith et al. (2011) noted that the relationship between ProM and the Big Five factors was found in only one out of three studies of agreeableness, one out of four studies of neuroticism, and four out of five studies of conscientiousness. In turn, Smith et al. concluded that “conscientiousness plays at least a small role in determining PM (ProM) performance on both laboratory and naturalistic tasks” (p. 114).

Reviewing several recent studies on the relationship between ProM and Big Five personality factors reveals several methodological issues that may explain the inconsistency in findings. First, ProM has been investigated by using both objective task measures as well as self-report measures of ProM forgetting. Yet, it has been demonstrated that several of the self-report measures of ProM currently in use [e.g., Prospective and Retrospective Memory Questionnaire (PRMQ), Smith et al., 2000; Prospective Memory Questionnaire (PMQ), Hannon et al., 1995] lack convergent validity and show no or only weak correlations with performance on objective ProM tasks even when ProM is measured by reliable continuous measures (Uttl and Kibreab, 2011). Importantly, these self-report measures also lack divergent validity from self-report measures of retrospective memory (Uttl and Kibreab, 2011). To illustrate, the prospective and retrospective memory subscales of the PRMQ are highly correlated, with most of the reported correlations above 0.70 and often close to 0.80 (Mäntylä, 2003; Rönnlund et al., 2008; Macan et al., 2010). In contrast, objective measures of ProM and retrospective memory show only small correlations, often between 0.10 and 0.30 (Uttl et al., 2001; Uttl, 2006; Cuttler and Graf, 2007; Uttl and Kibreab, 2011). Thus, self-reports of ProM should not be considered valid measures of ProM ability (Uttl and Kibreab, 2011).

Second, performance on objective ProM measures is typically assessed using binary success/failure measures, even though the underlying ability is continuous and most likely normally distributed. One natural consequence of this coarse measurement of ProM ability is that prospective measures are imprecise and unreliable, and in turn, correlations between these binary success/failure measures of ProM and measures of other constructs are artificially attenuated (Graf and Uttl, 2001; Uttl, 2008, 2011). Dichotomous measures substantially underestimate the true population correlation between ProM and other constructs and, moreover, such underestimation is more severe as the proportion of successes to failures is more extreme (Uttl, 2008).

Third, a large proportion of ProM studies using objective measures also suffer from severe ceiling effects where large proportions of participants obtain perfect or nearly perfect scores (Uttl, 2005, 2008). In turn, these ceiling effects artificially reduce the magnitude of correlations that one may obtain between the ceiling-limited ProM scores and measures of other constructs such as personality. To illustrate, Uttl (2008) reviewed over 25 years of research on age declines in ProM and demonstrated empirically that the size of age declines reported in various studies was strongly correlated (*r* = 0.67) with the degree to which performance was limited by ceiling effects. When the ProM task was easy, older adults performed at the ceiling, and younger adults could thus not demonstrate their superior ProM ability, leading researchers to report that ProM did not decline with aging (rather then reporting their inability to measure any possible declines). In contrast, when the ProM task was more difficult, older adults performed below the ceiling, age declines were allowed to emerge, and researchers reported that ProM declined with aging.

Fourth, although the distinction between episodic ProM and vigilance/monitoring is widely acknowledged, it is rarely made explicit in the ProM literature and it is necessary to carefully examine the method section of each study to determine if a particular study investigated episodic ProM, vigilance/monitoring, or habitual ProM (Uttl, 2008). It is possible that personality factors such as conscientiousness, extroversion, and agreeableness could play a larger role in vigilance/monitoring vs. episodic ProM.

Finally, personality factors may play a larger role in ProM assessed in naturalistic vs. laboratory settings and in time vs. event cued ProM (EC ProM) tasks. To illustrate, conscientious people may use external reminders in naturalistic but not laboratory settings to help them remember to carry out plans (Uttl and Kibreab, 2011). Similarly, personality factors may play a larger role in time-cued tasks than in event cued tasks because of greater opportunities to set up external reminders for specific times vs. events. Previous studies have not usually distinguished between these various subdomains of ProM when assessing the impact of personality factors; thus, this could be one reason why the results of the studies to date have been inconsistent.

The present study had three major aims. The first aim was to systematically review the previous research on relationships between ProM and personality. The second aim was to examine the relationships between ProM and the Big Five personality factors using newly developed reliable continuous measures of ProM (Graf et al., 2002; Uttl, 2006; Uttl and Kibreab, 2011) and multiple measures of personality. The third aim was to examine the relationship between ProM and other individual difference variables including verbal intelligence and retrospective memory.

## Study 1

The objectives of this study were to systematically review the previous research on the relationship between ProM and personality, distinguishing between objective measures of ProM and self-report measures of ProM. Moreover, we grouped studies by the various subdomains of ProM (e.g., vigilance, episodic ProM, habitual ProM) and study setting (e.g., laboratory, natural). For each study, we also recorded the number of methodological features that may influence the size of the reported relationships, including presence of ceiling or floor effects, calculation of correlations, and other measures of associations over data aggregated across groups, reporting performance levels for both ProM and personality measures, and reporting of reliabilities of both prospective and personality measures. For example, if ProM measures are frequently afflicted by ceiling effects, as is common in studies of ProM and aging (Uttl, 2008, 2011), and if reliabilities of prospective measures are low (Graf and Uttl, 2001; Kelemen et al., 2006; Uttl, 2008), correlations between ProM and personality measures would be artificially reduced and possibly not detectable.

### Method

#### Studies included in meta-analysis

**Figure 1:**
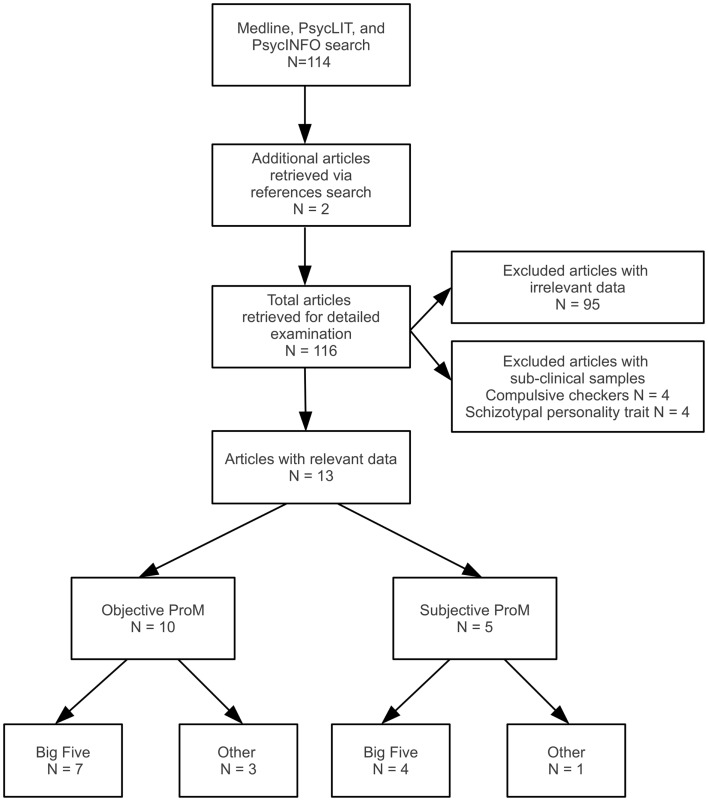
**Flowchart of the search strategy and selection of studies included in the review and meta-analysis**.

Figure 1 shows the search for relevant studies which proceeded in several steps. First, the PsycINFO, MEDLINE, and PsycARTICLES databases were searched from the earliest available date to the end of September 2012 for the following two sets of terms: (1) “prospective memory” and “memory for intentions,” and (2) “personality,” “openness,” “conscientiousness,” “extroversion,” “extraversion,” “agreeableness,” “neuroticism,” “Type A,” “state oriented,” “action oriented,” “Five Factor,” “Big Five,” “Cattell,” “NEO,” “BFI,” “International Personality Item Pool (IPIP),” and “MMPI.” The terms in each of the two sets were combined with OR to obtain all articles on ProM and personality including named personality factors. The two resulting sets were combined with AND. Second, the references in all relevant articles, book chapters, and theses, retrieved by any method, were examined for potentially relevant articles and the identified articles were hand searched for relevance.

To be included in the review, a study had to report correlations or other measures of association (e.g., regression results, mean differences) between objective or self-report measures of ProM successes or failures and personality factor(s); be written in English; and be based on normal healthy adults. Tasks were considered objective ProM tasks if they required participants to perform some action in the future without any prompting from experimenter.

#### Recorded variables

For each study, the recorded variables included authors; year of publication; study setting (laboratory vs. naturalistic); ProM subdomain (ProM proper, Habitual ProM, vigilance/monitoring); ProM cue type (event, time); study condition; personality test used [e.g., NEO FFI, NEO personality inventory revised (NEO PI-R), BFI]; ProM task; correlations (and other measures of association) between ProM and personality factors; participants (e.g., students, adults); participants’ age range; and methodological issues (e.g., presence of ceiling effects, correlations calculated across groups performing differently on ProM and personality tasks).

##### Objective vs. self-report measures of ProM

Tasks where participants had to respond to the ProM cue with some action were classified as objective ProM tasks whereas tasks where participants were asked to report on the frequency of their ProM failures were classified as self-report tasks.

##### ProM proper, vigilance/monitoring, and habitual ProM

Each ProM task was classified as measuring episodic ProM (eProM), vigilance/monitoring, or habitual ProM (Graf and Uttl, 2001; Uttl, 2008). Tasks that included a time delay or intervening task(s) between ProM instructions and commencement of an ongoing task were classified as measuring ProM proper whereas tasks that included no delay between instructions and the ongoing task were classified as measuring vigilance/monitoring.

##### Event-cued vs. time-cued ProM

Each ProM task was classified as measuring EC ProM if the task required a response to an event cue and as measuring time cued ProM if it required a response at a specific time.

##### Laboratory vs. naturalistic setting

Tasks performed under controlled laboratory conditions were classified as performed in laboratory settings whereas tasks performed by participants as part of their daily activities were classified as performed in naturalistic settings.

#### Meta-analysis methodology

Effect sizes – *r*s – were obtained for each reported relationship between each ProM measure and each personality factor. If a study reported correlations between two ProM measures of the same kind (e.g., two self-reports, two measures of the same ProM subdomain), we calculated the average of the two effect sizes and used the average in the meta-analysis (Hunter and Schmidt, 2004).

### Results

The search described in Figure 1 identified 13 articles that reported relationships between ProM measures and personality factors. Seven articles reported the relationships between objective measures of ProM and the Big Five and four reported the relationships between self-report measures of ProM and the Big Five. In contrast, only three articles reported the relationships between objective measures of ProM and other, non-Big Five personality factors and only one article reported the relationships between the self-report measures of ProM and other non-Big Five personality factors.

#### Objective measures of ProM and personality

Table 1 shows the relationships between the objective measures of ProM and the Big Five personality factors by task setting (laboratory vs. natural) and cue type (event vs. time). The number of studies for all but EC ProM assessed in laboratory settings was too small and precluded the possibility of any formal meta-analysis of the data. For EC ProM assessed in laboratory settings, only six studies (two assessed vigilance and four assessed episodic ProM) were available for meta-analysis and only five of them reported correlations between ProM measures and all the Big Five factors. In addition, Table 1 shows effect size indexes available for each ProM and personality relationship (e.g., “cor” for zero order correlation, “hr/beta” for hierarchical regression analysis beta coefficients); whether or not means, SD, and reliabilities were reported for both ProM and personality measures; and methodological features that may invalidate the findings (e.g., ceiling effects, aggregate groups analysis).

**Table 1:** **Relationship between objective measures of prospective memory and big five personality factors**.

Reference	*N*	Participants	Personality measures	ProM task	Sub domain	Effect index^1^	*O*	*C*	*E*	*A*	*N*	Pers. *M*/SD^2^	Pers. *r_xx_*^3^	ProM *M*/SD^4^	ProM *r_xx_*^5^	Notes^6^
**LABORATORY/EVENT CUED**																
Guynn (2001)	48	Students	NEO FFI	STM task w/cues	Vigilance	cor				−**0.31**	**0.30**	n	n	y	n	ag, *c*
Salthouse (2004)	330	18–89 years old	NEO FFI	Composite	Vigilance	cor	0.08	0.13	0.13	**0.16**	−0.08	y	y	y	y	ag, *c*
Breneiser (2007)	189	Students	NEO FFI	Synonyms	eProM	cor	**0.22**	0.00	−0.10	−0.01	−0.08	n	n	y	n	ag
Cuttler and Graf (2007)	141	18–81 years old	NEO PI-R	Questionnaire	eProM	cor	0.06	0.07	0.12	**0.18**	−0.04	n	y	y	n	ag, *c*
Cuttler and Graf (2007)	141	18–81 years old	NEO PI-R	Plug-in-the-phone	eProM	cor	0.12	0.00	0.03	−0.03	**0.28**	n	y	y	n	ag
Smith et al. (2011)	413	Students	BFI	Lexical decision	eProM	cor	0.04	**0.10**	0.00	0.08	−0.01	y	y	y	y	
Uttl and Kibreab (2011)	176	Students	NEO FFI	EC ProM/*C*	eProM	cor	**0.14**	**0.13**	−0.07	**0.19**	−**0.20**	y	y	y	y	
**NATURALISTIC/EVENT CUED**																
Pearman and Storandt (2005)	85	56–94 years old	NEO PI-R	Two tasks	eProM	cor		0.20			−0.08	y	n	n	n	
**NATURALISTIC/TIME CUED**																
Cuttler and Graf (2007)	141	18–81 years old	NEO PI-R	Confirmation-call	eProM	cor	−0.11	**0.21**	−0.14	0.14	−0.13	n	y	y	n	ag
Uttl and Kibreab (2011)	240	Students	NEO FFI	TC ProM	eProM	cor	0.05	0.12	−0.09	0.10	−0.05	y	y	y	y	

*Note. *O*, Openness; *C*, Conscientiousness; *E*, Extroversion; *A*, Agreeableness; *N*, Neuroticism; ^1^cor, zero order correlation; hr/beta, hierarchical regression/beta values; loading, factor loading; ^2^Were *Ms* and SDs for personality measures reported? y, yes; n, no; ^3^Were reliabilities of personality measures reported? y, yes; n, no; ^4^Were *Ms* and SDs for ProM measures reported? y, yes; n, no; ^5^Were reliabilities of ProM measures reported? y, yes; n, no. ^6^ag, aggregate group; *c*, ceiling effects; NEO FFI (Costa and McCrae, 1992); NEO PI-R (Costa and McCrae, 1992); BFI (John and Srivastava, 1999). Bold indicates p < 0.05*.

**Figure 2:**
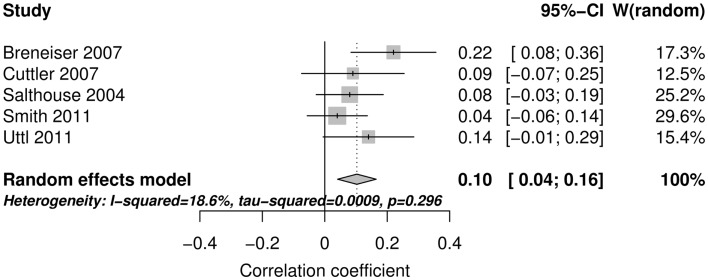
**The forest plot and random effect model meta-analysis for openness**.

**Figure 3:**
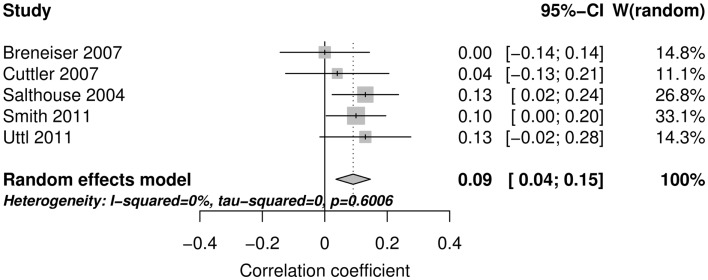
**The forest plot and random effect model meta-analysis for conscientiousness**.

**Figure 4:**
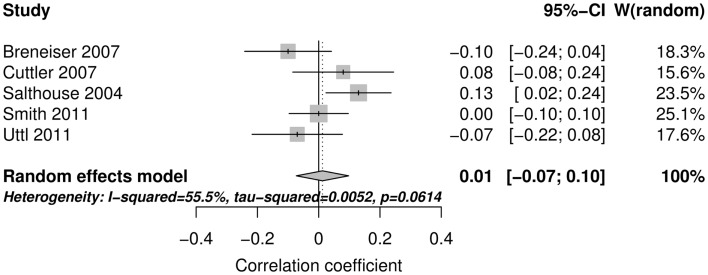
**The forest plot and random effect model meta-analysis for extroversion**.

**Figure 5:**
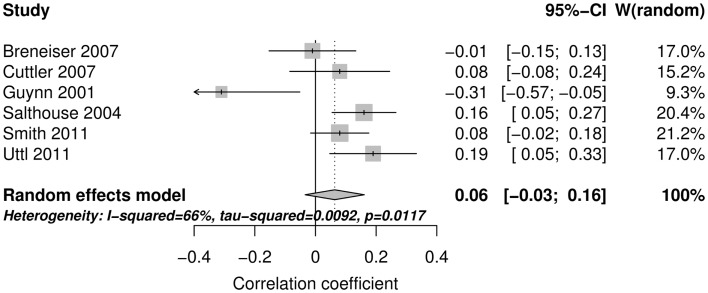
**The forest plot and random effect model meta-analysis for agreeableness**.

**Figure 6:**
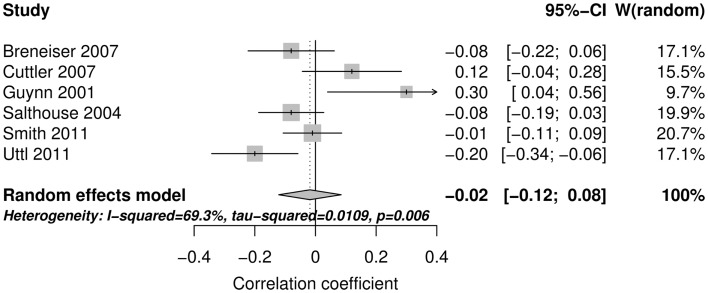
**The forest plot and random effect model meta-analysis for neuroticism**.

Figure 2 shows the forest plot and random effect model meta-analysis for openness. The forest plot highlights that all five studies reported positive correlations between ProM and openness. The meta-analysis resulted in a small but statistically significant effect size of *r* = 0.10 with 95% CI = (0.04, 0.16) (number of studies *k* = 5, individual study *n*s ranging from 141 to 413). The effects were relatively homogeneous (*I*^2^ = 18.6%, *T*^2^ < 0.001). Figure 3 shows that the random effect model meta-analysis revealed a small but statistically significant association between ProM and conscientiousness [*r* = 0.09; 95% CI = (0.04, 0.15); *I*^2^ = 0%, *T*^2^ = 0; *k* = 5, individual study *n*s ranging from 141 to 413]. Figure 4 shows the results for extroversion. The overall strength of the association between ProM and extroversion was negligible and not statistically significant [*r* = 0.01; 95% CI = (−0.07, 0.10); *I*^2^ = 55.5%, *T*^2^ = 0.005; *k* = 5, individual study *n*s ranging from 141 to 413]. Figure 5 shows the random effect meta-analysis results for agreeableness. It indicates that the correlation between ProM and agreeableness was small and not statistically significant [*r* = 0.06; 95% CI = (−0.03, 0.16); *I*^2^ = 66%, *T*^2^ = 0.01; *k* = 6, individual study *n*s ranging from 48 to 413]. However, Guynn’s (2001) study appears to be an outlier from the rest of the effect sizes. After excluding this one study, the remaining effect sizes became homogenous (*I*^2^ = 23.7%, *T*^2^ = 0.001) and the correlation between ProM and agreeableness became statistically significant [*r* = 0.10, 95% CI = (0.04, 0.17); *k* = 5, individual study *n*s ranging from 141 to 413]. Figure 6 shows the results for neuroticism. The overall strength of the association was negligible and not statistically significant [*r* = −0.02; 95% CI = (−0.12, 0.08); *I*^2^ = 69.3%, *T*^2^ = 0.011; *k* = 6, individual study *n*s ranging from 48 to 413]. Removing Guynn’s study improved homogeneity (*I*^2^ = 58.2%, *T*^2^ = 0.006) but the overall strength of the association remained negligible and not statistically significant [*r* = −0.05, 95% CI = (−0.14, 0.03); *k* = 5, individual study *n*s ranging from 141 to 413].

Table 2 shows the relationships between the objective measures of ProM and other non-Big Five personality factors by task type and cue type. For each study and ProM task, the table lists personality factors correlated with the ProM task, whether each relationship was statistically significant and whether it was positive or negative. Statistically significant correlations/relationships are indicated by either plus (positive relationships) or minus (negative relationships) signs preceding the specific personality factor name. Fractions such as “1/5” indicate how many of the investigated relationships were statistically significant (e.g., 1/5 indicates that out of 5 ProM and personality correlations only one was found statistically significant).

**Table 2:** **Relationship between objective measures of ProM and other personality factors**.

Reference	*n*	Participants	Personality measures	ProM task	Sub domain	Effect index^1^	Personality factors^2^	Pers. *M*/SD^3^	Pers. *r_xx_*^4^	ProM *M*/SD^5^	ProM *r_xx_*^6^	Notes^7^
**LABORATORY/EVENT CUED**												
Arana et al. (2008)	157	Students	16PF-5	Glasses	Vigilance	Hr/beta	Specific (1/5; +reasoning)	y	y	y	n	
Arana et al. (2008)	157	Students	16PF-5	Underline spec word	eProM	Hr/beta	Specific (1/5; +rule-consciousness) global (1/5; +self-control)	y	y	y	n	
Cuttler and Graf (2007)	141	18–81 years old	NEO PI-R	Questionnaire Task	eProM	Cor	Perfectionism (1/3; +socially prescribed)	n	y	y	n	ag, *c*
Cuttler and Graf (2007)	141	18–81 years old	NEO PI-R	Plug-in-the-Phone Task	eProM	Cor	Perfectionism (0/3)	n	y	y	n	ag
Rönnlund et al. (2011)	255	60–94 years old	TCI	Reminding task	eProM	Hr/beta	Harm avoidance self-directedness	n	n	n	y	ag
Searleman (1996)	80	Students	JAS POI	Report favorite show	eProM		+Type A self-actualization	n	n	y	n	
Searleman (1996)	60	Students	JAS	Remind phone call	eProM	Cor	+Type A	n	n	y	n	*c*
			Kline Ai3				Self-actualization	
			POI				Obsessive compulsive	
			Self-monitoring				Self-monitoring	
**LABORATORY/TIME CUED**												
Arana et al. (2008)	157	Students	16PF-5	Sign every 5 min	eProM	Hr/beta	Global (0/5)	y	y	y	n	
**NATURALISTIC/TIME CUED**												
Cuttler and Graf (2007)	141	18–81 years old	NEO PI-R	Confirmation-Call Task	eProM	Cor	Perfectionism (1/3; +socially prescribed)	n	y	y	n	ag
Searleman (1996)	80	Students	JAS	Leave a message	eProM		Type A	n	n	y	n	*c*
			POI				self-actualization	
Searleman (1996)	60	Students	JAS	Date and time a card	eProM	Cor	Type A	n	n	y	n	
			Kline Ai3				Self-actualization	
			POI				Obsessive compulsive	
			Self-monitoring				Self-monitoring	

*Note. *O*, Openness; *C*, Conscientiousness; *E*, Extroversion; *A*, Agreeableness; *N*, Neuroticism; ^1^cor, zero order correlation; hr/beta, hierarchical regression/beta values; loading, factor loading; ^2^The column lists personality factors investigated in each study condition beyond the Big Five. The “+” sign in front of the factor indicates the relationship between ProM and the specific personality factor was statistically significant and positive and the “−” sign indicates that the relationship was statistically significant and negative. The fractions such as 1/3 indicate that out of 3 personality factors examined only one was significantly related to ProM. ^3^Were *M*s and SDs for personality measures reported? y, yes; n, no; ^4^Were reliabilities of personality measures reported? y, yes; n, no; ^5^Were *M*s and SDs for ProM measures reported? y, yes; n, no; ^6^Were reliabilities of ProM measures reported? y, yes; n, no. ^7^ag, aggregate group; *c*, ceiling effects; 16PF-5 (Cattell et al., 1993); NEO FFI (Costa and McCrae, 1992); JAS (Krantz et al., 1974); POI (Jones and Crandall, 1986); Kline Ai3 (Kline, 1968)*.

The studies listed in Table 2 used small to moderate sample sizes (60–255 participants), a variety of personality measures, a variety of effect size indexes (correlations, beta weights from hierarchical regression analyses), and examined relationships between ProM and a wide variety of personality traits. As shown in Table 2, the most common finding was that personality traits did not correlate with ProM measures.

#### Self-reports of ProM and personality

Table 3 shows the relationship between self-reports of ProM failures and personality. Only two studies reported the relationship between ProM and each of the Big Five factors: one reported the relationship between self-reports of ProM and extroversion, and one reported the relationship between self-reports of ProM and neuroticism. Finally, one study reported the relationship between self-reported ProM failures and other personality traits: harm avoidance and self-directedness. The studies used small to large samples (56–1291 participants), a variety of self-reports of ProM failures, and a variety of effect size indexes (correlations, beta weights, loadings).

**Table 3:** **Relationship between self-reports of ProM failures and big five personality factors**.

Reference	*n*	Participants	Personality measures	ProM self-report	Effect index^1^	*O*	*C*	*E*	*A*	*N*	Other personality factors^2^	Pers. M/SD^3^	Pers. r_xx_^4^	ProM M/SD^5^	ProM r_xx_^6^	Notes^7^
**BIG FIVE**																
Gondo et al. (2010)	459	Students	NEO FFI	PRMQ	Hr/beta	0.05	−**0.35**	0.07	0.01	**0.13**	+ Perfectionism	y	y	y	y	
Gondo et al. (2010)	1291	50–69 years old	NEO FFI	PRMQ	Hr/beta	0.06	−**0.15**	0.04	−0.07	**0.29**	Perfectionism	y	y	y	y	
Gondo et al. (2010)	860	70–79 years old	NEO FFI	PRMQ	Hr/beta	−0.02	−**0.18**	0.03	−0.01	**0.25**	Perfectionism	y	y	y	y	
Heffernan and Ling (2001)	56	Students	EPQR	PMQ LTE	Cor/msd			−**0.19**				n	n	y	y	
Heffernan and Ling (2001)	56	Students	EPQR	PMQ STH	Cor/msd			−**0.45**				n	n	y	y	
Uttl (2011)	240	Students	NEO FFI	PMQ LTE	Cor	−0.04	−**0.33**	0.04	−0.01	**0.16**		y	y	y	y	
Uttl (2011)	240	Students	NEO FFI	PMQ STE	Cor	−0.03	−**0.25**	0.12	−0.02	0.08		y	y	y	y	
Uttl (2011)	240	Students	NEO FFI	PMQ IC	Cor	−0.10	−**0.34**	0.02	−0.05	**0.23**		y	y	y	y	
Uttl (2011)	240	Students	NEO FFI	PRMQ	Cor	0.10	−**0.30**	−0.13	−0.02	**0.26**		y	y	y	y	
Uttl (2011)	240	Students	NEO FFI	MemQ ProM	Cor	0.12	−**0.50**	−0.05	0.03	**0.26**		y	y	y	y	
Uttl (2011)	240	Students	NEO FFI	CAPM/A IADL	Cor	0.11	−**0.37**	−0.09	0.04	**0.18**		y	y	y	y	
Uttl (2011)	240	Students	NEO FFI	CAPM/A BADL	Cor	−0.02	−**0.32**	−0.03	0.04	**0.13**		y	y	y	y	
Uttl (2011)	240	Students	NEO FFI	TCPMQ Freq	Cor	0.03	−**0.42**	0.00	0.02	**0.29**		y	y	y	y	
Zimprich et al. (2011)	336	66–81 years old	NEO FFI	PRMQ/modified	Loading					**0.13**		n	n	n	y	
**OTHER**																
Rönnlund et al. (2011)	255	60–94 years old	TCI	PRMQ	Hr/beta						Harm avoidance +self-directedness	n	n	n	y	ag

*Note. *O*, Openness; *C*, Conscientiousness; *E*, Extroversion; *A*, Agreeableness; *N*, Neuroticism; ^1^cor, zero order correlation; cor/msd, correlations obtained from the mean differences; hr/beta, hierarchical regression/beta values; loading, factor loading; ^2^The column lists personality factors investigated in each study condition beyond the Big Five. The “+” sign in front of the factor indicates the relationship between ProM and the specific personality factor was statistically significant and positive and the “−” sign indicates that the relationship was statistically significant and negative. The fractions such as 1/3 indicate that out of 3 personality factors examined only one was significantly related to ProM. ^3^Were *Ms* and SDs for personality measures reported? y, yes; n, no; ^4^Were reliabilities of personality measures reported? y, yes; n, no; ^5^Were *Ms* and SDs for ProM measures reported? y, yes; n, no; ^6^Were reliabilities of ProM measures reported? y, yes; n, no. ^7^ag, aggregate group; *c*, ceiling effects; NEO FFI (Costa and McCrae, 1992); EPQR (Eysenk and Eysenk, 1991); PRMQ (G. Smith et al., 2000); PMQ (Hannon et al., 1995); MemQ (Uttl and Kibreab, 2011); CAPM (Roche et al., 2007); TCPMQ (Cuttler and Graf, 2009). Bold indicates p < 0.05*.

The scarcity of studies as well as failures to report zero order correlations among the measures precluded any formal meta-analyses (Hunter and Schmidt, 2004). However, correlations and beta weights reported in the three moderate to large sized studies (Gondo et al., 2010; Uttl and Kibreab, 2011; Zimprich et al., 2011) indicate that self-reported ProM failures are negatively correlated with conscientiousness, but positively correlated with neuroticism. Although Heffernan and Ling (2001) reported a negative association between extroversion and self-reported ProM failures, the results of other studies are inconsistent with this finding.

### Discussion

The current results indicate that openness, conscientiousness, and agreeableness are weakly related to performance on objective measures of EC ProM tasks assessed in laboratory settings, with *r*s ranging from 0.09 to 0.10. Thus, the failure to consistently find statistically significant relationships between ProM measures and openness, conscientiousness, and agreeableness in individual studies is not surprising. To detect a population correlation of 0.10, the necessary sample size is *n* = 782 for power = 0.80 and *n* = 1293 for power = 0.95. In contrast, to detect a population correlation of 0.20, the necessary sample size is *n* = 194 for power = 0.80 and *n* = 319 for power = 0.95. Thus, although some studies listed in Table 1 were powerful enough to detect 0.20 correlations with power approximately 0.80 or larger, none were powerful enough to detect 0.10 correlations between ProM and personality. Similarly, previous studies that examined the relationship between EC ProM in laboratory settings and other personality factors (e.g., Type A, perfectionism, self-actualization) were similarly underpowered, revealed a few statistically significant findings, and no consistency across the studies (see Table 2).

In contrast to objective measures of ProM, self-report measures of ProM failures were negatively correlated with conscientiousness and agreeableness. This conclusion rests principally on the results of the two large studies that examined the correlations between self-reported ProM failures and all of the Big Five factors (Gondo et al., 2010; Uttl and Kibreab, 2011), as we were unable to conduct a formal meta-analysis due to the small number of independent studies. However, this conclusion is strengthened by the observation that the same pattern of findings held across five different self-report measures of ProM failures used in the Uttl and Kibreab study. The findings that the self-report measures of ProM failures exhibit different patterns of correlations with the Big Five and that such correlations seem to be generally stronger is consistent with the findings that the self-report measures of ProM are not valid measures of ProM ability. The self-reports are influenced by a wide variety of other factors that are unlikely to influence performance in laboratory settings such as busyness (Martin and Park, 2003), how many activities and events respondents are involved in (Uttl and Kibreab, 2011), and how often and how many memory strategies and external aids they use (Uttl and Kibreab, 2011).

This review revealed a number of methodological issues that further complicate and limit the interpretation of previous findings as well as the current meta-analysis. First, the majority of studies investigating the relationship between objective measures of ProM and personality did not report reliabilities of their ProM measures and several did not report reliabilities of their personality measures. However, if we do not know the reliabilities of the measures, it is impossible to determine whether the lack of relationships and generally small correlations between ProM and personality measures are due to poor reliabilities of inadequate measures or whether they reflect the true strength of the associations between these abilities.

Second, in a number of studies, performance on ProM measures was limited by ceiling effects. In turn, the ceiling effects may have reduced observed correlations just like they did in many studies on ProM and aging. As noted in the introduction, Uttl (2008, 2011) found correlation −0.67 between the size of age declines and the degree of ceiling effects. When performance was severely limited by ceiling effects, researchers reported no age declines in ProM. In contrast, when performance was not limited by ceiling effects, researchers reported substantial age declines.

Third, a number of the studies listed in Table 1 calculated correlations between ProM and personality factors across all study participants even though participants belonged to different experimental and/or age groups that performed differently on ProM and/or personality measures. Accordingly, the reported correlations may reflect group differences rather than associations between ProM and personality. The reported correlations may be larger or smaller than the true correlations depending on the true within-group correlations as well as on the exact patterns of performance of various groups on the measures of ProM and personality (Kaplan and Saccuzzo, 2012).

Finally, the data accumulated to date do not allow us to make any conclusions about whether the relationship strength between ProM and personality depends on ProM subdomain, that is, whether such relationships are stronger in vigilance/monitoring vs. episodic ProM because only two studies examined the relationship between vigilance/monitoring and the Big Five factors.

## Study 2

The objectives of the study were to examine the relationship between episodic ProM, Big Five personality factors, verbal intelligence, and retrospective memory. To avoid some of the shortcomings of previous research, in the current study the key constructs were assessed by multiple reliable measures: (1) episodic ProM was assessed twice using reliable continuous measures of ProM (Graf et al., 2002; Uttl, 2006; Uttl and Kibreab, 2011), (2) the Big Five personality factors were assessed twice using NEO PI-R (Costa and McCrae, 1992) and International personality item pool NEO (IPIP NEO PI-R) (Goldberg et al., 2006), and (3) verbal intelligence was assessed by three different verbal knowledge measures (Shipley, 1940; Uttl and Kibreab, 2011).

### Method

#### Participants

Participants were 378 undergraduate student volunteers, 73.9% females and 26.1% males (age *M* = 21.2 years, SD = 4.9 years). The majority of participants spoke English as their first language (88%). The study was approved by Mount Royal University Human Research Ethics Board and all participants gave prior written consent to participate in the study. The study took approximately 2 h and each participant received two course participation credits.

#### Design

In addition to examining the relationship between ProM, personality, and individual differences, the study was designed to examine the effects of delays on ProM. For this purpose, there were two between subject factors: Instructions to Ongoing Task Delay (I-O Delay) (0 vs. 7 min) and Ongoing Task to (the first) Cue Delay (O-C Delay) (0 vs. 15 min). However, for the purposes of this study, all delay conditions were collapsed into the episodic ProM proper condition and the no-delay condition was used to examine the relationship between vigilance/monitoring, personality, and individual differences.

#### Measurement instruments

As part of a larger study, participants were administered several objective tests of ProM and retrospective memory, three measures of verbal intelligence, and two personality questionnaires. These tests are described below.

##### Continuous measures of event cued ProM (EC PromM/C)

The continuous measures of EC ProM developed for this study were patterned after continuous measures previously used by Uttl and his colleagues (Graf et al., 2002; Uttl, 2006; Uttl and Kibreab, 2011). Participants’ ProM was assessed on two occasions within the session to allow assessment of test-retest reliability. For each assessment, participants were instructed to circle any and all occurrences of the ProM cue – the word “close” (first assessment) and the word “above” (second assessment) – as they worked through the session. The specific instructions for the first assessment were:

We want to examine your ability to do something in future. Thus, if you encounter the word *close* at any point during this experiment, please circle it. You will not be reminded again but it is important that you circle any and all occurrences of the word *close*. Please copy the following sentence below in your hand writing so that we are sure you did not miss these instructions: I am to circle all occurrences of the word *close*.

The instructions for the second assessment were identical except that the word *close* was replaced with the word *above*. Following these instructions, participants worked through the tasks.

On each assessment, 11 ProM cues were embedded within a personality inventory designed to measure the Big Five and NEO PI-R facets. On the first assessment, the cues appeared within the 300-item IPIP NEO (Goldberg et al., 2006) extended with 19 fillers and 11 items that included ProM cues. On the second assessment, the cues appeared within the 240 NEO PI-R (Costa and McCrae, 1992), extended with 79 fillers and 11 items that included ProM cues. Both fillers and items containing ProM cues were drawn from the IPIP database. Thus, on each assessment, participants encountered the cues within a 23-page personality inventory. The first page contained the standard instructions for the personality inventory and each of the subsequent 22 pages contained 15 personality items. In the 0 min O-C delay condition, the first cue appeared on page 2, immediately after the personality inventory instructions, and subsequent cues appeared on pages 3 to 12, one per page. In the 15 min O-C delay condition, the first cue appeared on page 13, and subsequent cues appeared on pages 14–23, one per page.

Critically, the ProM cue became progressively larger and more intrusive on each successive page. The smallest cue size was 12-pt font (identical to the surrounding text font size) and the largest cue size was 28-pt font. If a participant detected the very first cue, he or she obtained a score of 11. If the participant detected the last cue, he or she obtained a score of 1, and if the participant did not detect any cues, he or she obtained a score of 0. Thus, each participant’s test score was determined solely by the first cue to which the participant responded.

##### Binary success/failure measures of event cued ProM (EC ProM/B)

To compare performance on binary vs. continuous measures, for each ProM assessment described above, we also calculated a binary success/failure ProM measure based solely on participants’ performance on the first shown ProM cue. If a participant responded to the very first cue, he or she obtained a score of 1. If the participant did not respond to the first cue, he or she received a score of 0.

##### NEO Personality Inventory Revised

The NEO PI-R (Costa and McCrae, 1992) is a 240-item self-report personality inventory measuring five personality domains: neuroticism, extroversion, openness to experience, agreeableness, and conscientiousness (Big Five). Participants rated each item using a 5-point Likert scale (1, Strongly disagree; 2, Disagree; 3, Neutral; 4, Agree; 5, Strongly Agree). Each of the five personality dimensions is assessed using 48 items, and, according to the Costa and McCrae scoring scheme, each dimension’s score can range from 48 to 240. However, for ease of interpretation, we calculated the scores for each dimension as the average across the relevant items, with personality dimension scores ranging from 1 to 5.

##### International Personality Item Pool NEO

The IPIP NEO (Goldberg et al., 2006) is a 300-item self-report personality inventory designed to measure the Big Five and the NEO PI-R facets using items from the IPIP. Participants rated how accurately each statement describes them using the following response scale: 1, Very inaccurate; 2, Moderately inaccurate; 3, Neither inaccurate nor accurate; 4, Moderately accurate; and 5, Very accurate. Similarly to the NEO PI-R, we calculated each participant’s personality dimension scores as the average across the relevant items. Thus, the scores ranged from 1 to 5 for each of the Big Five.

##### Verbal Learning Test Unrelated 20

The Verbal learning test unrelated 20 (VLT/U20) (Uttl, 2006) is a measure of explicit episodic RetM patterned after the Rey Auditory Verbal Learning Test (Strauss et al., 2006). The test consists of a series of three study-test trials with a list of 20 semantically unrelated words. On each trial, a participant listened to a list of 20 words read by an experimenter and was required to write down as many words as he/she could remember, in any order, after the experimenter had completed reading the list. For each trial, participants were given 90 s for recall. Thus, the scores on each trial as well as the average of the three trials score could range from 0 to 20.

##### Words/A40 and Words/B40

The Words/A40 and Words/B40 are 40-item multiple choice tests designed to assess examinees’ verbal knowledge (Uttl, 2006). Each item consists of a target word and four other words out of which one word is similar in meaning. Each item is scored as correct (1 point), incorrect (0 points), or not answered (0.25 points to correct for failure to guess). The test score is the proportion of items correctly answered. The only difference between Words/A40 and Words/B40 is that the two tests use different set of words.

##### Shippley’s Institute for Living Scale Vocabulary Test

The Shippley’s institute for living scale (SILS) Vocabulary (Shipley, 1940) is a 40-item multiple choice test designed to assess examinees’ vocabulary. Examinees’ are asked to identify which word out of four is the most similar in meaning to the target word. For purposes of this study, the test score is the proportion of items correctly answered.

#### Procedure

Participants were tested in small groups, seated widely separated in a small classroom, in a single session lasting about 2 h. First, participants provided written informed consent and basic demographic information (age, gender, whether their first language was English). Second, they completed a set of timed tasks including VLT/U20, a measure of retrospective episodic memory. And third, they completed the critical set of tasks described above: ProM Instructions 1, Words/A40, IPIP NEO PI-R w/embedded ProM cues, Words/B40, ProM Instructions 2, SILS Vocabulary, and NEO PI-R w/embedded ProM cues (the order of Words/A40 and IPIP NEO PI-R and the order of SILS Vocabulary and NEO PI-R is shown for the I-O Delay condition; the order was switched in the no I-O Delay condition). Participants completed this last set of tasks at their own pace, placing each completed page on the desk directly behind them (this prevented them from going back and circling cues they may have missed previously).

### Results

#### Data screening

The data were screened for univariate outliers defined as scores falling 1.5 interquartile ranges below the 25th percentile or above the 75th percentile. Less than 3% of data values were univariate outliers. The influence of outliers was reduced by replacing them with corresponding outlier caps (i.e., a value 1.5 interquartile range either below the 25th percentile or above the 75th percentile, as appropriate).

#### Binary vs. continuous measures of ProM

Table 4 shows the means, SD, and reliabilities for composite measures (averages) of the two ProM assessments, for binary as well as for continuous measures. The data are shown separately for vigilance/monitoring (i.e., the condition with no delay between ProM instructions and the appearance of the first ProM cue) and for episodic ProM (i.e., the conditions with a delay between ProM instructions and the appearance of the first ProM cue). Consistent with theoretical expectations as well as prior findings, performance increased from the first to the second assessment on both binary and continuous measures of episodic ProM but no such changes in performance were observed on binary or continuous measures of vigilance/monitoring.

**Table 4:** **Descriptive statistics and reliabilities**.

	Vigilance/monitoring (*n* = 95)			Episodic ProM (*n* = 283)		
	*M*	SD	α	*M*	SD	α
ProM 1/B	0.55	0.50		0.21	0.41	
ProM 2/B	0.59	0.49		0.36	0.48	
ProM/B	0.57	0.40	0.43	0.28	0.35	0.35
ProM 1/C	10.31	0.89		8.87	1.88	
ProM 2/C	10.23	1.12		9.70	1.28	
ProM/C	10.27	0.85	0.60	9.28	1.39	0.66
VLT/U20 A1	7.29	1.81		7.21	1.76	
VLT/U20 A2	10.72	2.24		10.75	2.47	
VLT/U20 A3	13.18	2.72		13.22	2.74	
VLT/U20	10.41	2.08	0.87	10.33	2.12	0.86
Words/A40	0.56	0.17	0.83	0.58	0.18	0.84
Words/B40	0.49	0.15	0.79	0.50	0.15	0.79
SILS vocabulary	0.64	0.13	0.84	0.64	0.12	0.80
IPIP openness	3.53	0.41	0.90	3.49	0.35	0.85
IPIP conscientiousness	3.49	0.49	0.94	3.48	0.42	0.92
IPIP extroversion	3.52	0.44	0.92	3.53	0.45	0.93
IPIP agreeableness	3.62	0.40	0.90	3.54	0.43	0.91
IPIP neuroticism	2.99	0.52	0.94	2.92	0.50	0.93
NEO openness	3.45	0.41	0.87	3.43	0.36	0.83
NEO conscientiousness	3.26	0.50	0.93	3.29	0.42	0.90
NEO extroversion	3.53	0.43	0.88	3.53	0.44	0.90
NEO agreeableness	3.50	0.39	0.82	3.39	0.43	0.90
NEO neuroticism	3.05	0.49	0.92	2.98	0.48	0.92

*Note. α, Cronbach’s α*.

As expected, the test-retest reliabilities of the binary measures were substantially lower than the test-retest reliabilities of the continuous measures. Accordingly, the Cronbach’s alpha of the two measures composites were higher for the continuous vs. binary measures (vigilance/monitoring: 0.60 vs. 0.43; episodic ProM: 0.66 vs. 0.35). The reliabilities of the composites were respectable, 0.66 for episodic ProM and 0.60 for vigilance/monitoring.

#### Measures of personality, retrospective memory, and verbal intelligence

Table 4 also shows the means, SD, and reliabilities for measures of the Big Five, retrospective memory, and verbal intelligence, for vigilance/monitoring and episodic ProM. As indicated in the table, there were no differences in performance between the two vigilance/monitoring and episodic ProM conditions on any of these measures except on NEO PI-R agreeableness [Welsh *t*(173.56) = 2.28, *p* = 0.02)].

Table 4 also shows that the reliabilities of the Big Five measures were high, for both IPIP and NEO PI-R, ranging from 0.85 to 0.94 for IPIP and from 0.82 to 0.93 for NEO PI-R. Moreover, as shown in Table 5, the correlations between IPIP and NEO PI-R measures were also high, 0.80–0.85 for openness; 0.87–0.88 for conscientiousness; 0.82–0.88 for extroversion; 0.85–0.87 for agreeableness; and 0.86–0.87 for neuroticism. The reliabilities of measures of retrospective memory and verbal intelligence were similarly high (see Table 4).

**Table 5:** **Correlations matrix**.

	1	2	3	4	5	6	7	8	9	10	11	12	13	14	15
**EPISODIC ProM (*n* = 283)**															
1. ProM/B															
2. ProM/C	**0.71**														
3. VLT/U20	0.07	0.07													
4. Words/A40	**0.18**	**0.22**	**0.26**												
5. Words/B40	**0.19**	**0.23**	**0.14**	**0.73**											
6. SILS/Voc	**0.12**	**0.17**	**0.23**	**0.66**	**0.64**										
7. NEO openness	0.05	0.09	0.08	**0.25**	**0.31**	**0.31**									
8. NEO conscientiousness	0.06	0.01	0.05	0.04	0.09	0.08	0.05								
9. NEO extroversion	−0.07	−0.01	0.06	−**0.13**	−**0.13**	−**0.13**	**0.29**	**0.20**							
10. NEO agreeableness	0.01	0.00	−0.11	0.09	**0.15**	0.11	**0.22**	**0.25**	0.06						
11. NEO neuroticism	−0.08	−0.04	−0.05	−0.08	−**0.14**	−0.07	−0.08	−**0.50**	−**0.33**	−**0.29**					
12. IPIP openness	0.04	0.04	−0.02	**0.15**	**0.17**	**0.15**	**0.80**	0.04	**0.26**	**0.20**	−0.05				
13. IPIP conscientiousness	0.05	−0.03	0.03	0.05	0.09	0.07	0.04	**0.87**	**0.12**	**0.32**	−**0.42**	0.08			
14. IPIP extroversion	−0.06	−0.03	0.03	−**0.15**	−**0.16**	−**0.17**	**0.25**	**0.12**	**0.88**	−0.05	−**0.32**	**0.29**	0.10		
15. IPIP agreeableness	−0.01	−0.03	−0.06	0.04	**0.12**	0.10	**0.26**	**0.24**	0.05	**0.87**	−**0.19**	**0.27**	**0.40**	−0.02	
16. IPIP neuroticism	−0.08	−0.04	−0.06	−**0.14**	−**0.16**	−**0.12**	−**0.13**	−**0.48**	−**0.38**	−**0.25**	**0.87**	−**0.13**	−**0.43**	−**0.41**	−**0.17**
**VIGILANCE/MONITORING (*n* = 95)**															
1. Vigilance/B															
2. Vigilance/C	**0.85**														
3. VLT/U20	0.14	0.16													
4. Words/A40	**0.30**	**0.33**	**0.27**												
5. Words/B40	**0.20**	**0.26**	0.18	**0.82**											
6. SILS/voc	**0.27**	**0.36**	**0.23**	**0.67**	**0.61**										
7. NEO openness	**0.21**	**0.23**	0.17	**0.48**	**0.34**	**0.39**									
8. NEO conscientiousness	0.14	0.12	**0.20**	**0.20**	0.11	0.16	0.07								
9. NEO extroversion	**0.29**	**0.27**	0.15	0.14	0.02	0.19	**0.34**	**0.28**							
10. NEO agreeableness	0.00	0.05	0.01	**0.21**	**0.27**	**0.28**	**0.27**	0.01	**0.21**						
11. NEO neuroticism	−0.10	−0.06	−0.09	−**0.21**	−0.16	−0.11	−0.11	−**0.51**	−**0.33**	−**0.32**					
12. IPIP openness	0.12	0.16	0.20	**0.32**	0.15	**0.27**	**0.85**	0.07	**0.27**	**0.24**	−0.07				
13. IPIP conscientiousness	0.06	0.06	**0.23**	0.15	0.07	0.12	0.02	**0.88**	**0.25**	0.07	−**0.38**	0.10			
14. IPIP extroversion	**0.21**	**0.22**	0.18	−0.02	−0.11	0.09	**0.24**	0.15	**0.82**	0.06	−**0.21**	**0.35**	**0.22**		
15. IPIP agreeableness	−0.04	0.07	0.11	**0.26**	**0.29**	**0.32**	**0.29**	0.07	0.17	**0.85**	−**0.26**	0.27	0.19	0.07	
16. IPIP neuroticism	−0.12	−0.10	−**0.20**	−**0.22**	−0.19	−0.13	−0.14	−**0.48**	−**0.34**	−**0.26**	**0.86**	−0.11	−**0.40**	−**0.26**	−0.15

*Note. Bold print, *p* < 0.05*.

#### Correlations between ProM and personality

Table 5 shows the correlations between ProM measures – episodic ProM and vigilance/monitoring – and the Big Five factors, for the continuous as well as for binary measures. Episodic ProM was not significantly correlated with any of the Big Five factors. In contrast, vigilance/monitoring was weakly correlated with extroversion, using both IPIP NEO and NEO PI-R and with openness. However, the correlation with openness reached statistical significance only when measured using NEO PI-R. Note, however, that the four reported correlations between openness and vigilance/monitoring were not statistically different from each other even though only two reached statistical significance.

#### Correlations between ProM, RetM, verbal intelligence, and personality

As shown in Table 5, correlations between retrospective memory and vigilance/monitoring and episodic ProM were weak and not statistically significant. In contrast, correlations between verbal intelligence measures and vigilance/monitoring and episodic ProM were small and statistically significant, consistent with prior findings.

### Discussion

The current study yielded the following key findings: First, event cued episodic ProM assessed in controlled laboratory conditions was not associated with any of the Big Five personality factors even though the study size was powerful enough to detect correlations of 0.16 with power equal to 0.80. Second, in contrast to episodic ProM, performance on event cued vigilance/monitoring was associated with extroversion and openness, that is, participants who scored higher on extroversion and openness scored better on ProM measures. Third, verbal intelligence, measured by three different measures, was associated with performance on both event cued episodic ProM and vigilance/monitoring tasks. And fourth, neither event cued episodic ProM nor vigilance/monitoring was associated with retrospective memory, consistent with a number of prior studies (Uttl et al., 2001; Graf et al., 2002; Cuttler and Graf, 2007; Zeintl et al., 2007; Uttl and Kibreab, 2011).

The finding that episodic ProM was not correlated with the Big Five personality factors is in general agreement with the results of the meta-analysis of prior research (Study 1) suggesting that the most strongly related personality factors – conscientiousness, openness, and agreeableness – correlate only 0.09–0.10 with ProM measures (even though the episodic ProM and personality correlations observed in Study 2 are not statistically significant, they are not statistically different from the estimates derived from the meta-analysis of prior research reported in Study 1, that is, the associated 95% confidence intervals include the estimates derived from the meta-analysis). As noted above, to detect such small correlations one would need to test 800 participants to obtain power equal to 0.80 and 1,300 participants to obtain power equal to 0.95. Critically, the lack of statistically significant correlations in our study cannot be attributed to unreliable measures, ceiling effects, or the group differences and methodological flaws undermining the validity and conclusions of many of the previous studies. We assessed ProM twice, used reliable continuous measures that were free of ceiling and floor effects, avoided confounding of ProM and personality correlations by group differences, and examined ProM and personality relationships in a large sample of undergraduate students.

The finding that, in contrast to event cued episodic ProM, event cued vigilance/monitoring is associated with openness and extroversion indicates that vigilance/monitoring is associated in part with different individual differences variables than episodic ProM. Openness and extroversion may help participants to orient in and engage with novel laboratory environments and novel tasks and to figure out strategies (e.g., keeping the plan in consciousness and looking out for cues) for succeeding on vigilance/monitoring tasks that may not be helpful in episodic ProM tasks, that is, in bringing the plan back to consciousness. However, it remains to be seen whether these associations will be replicated in future studies. Only two previous studies have examined the relationship between the Big Five personality factors and the vigilance/monitoring subdomain of ProM (Guynn, 2001; Salthouse et al., 2004) and both were confounded by group differences and ceiling effects (see Table 1).

Our data also show that both episodic ProM and vigilance/monitoring were associated with verbal intelligence measured by three verbal knowledge tests. These findings replicate several previous studies (Uttl and Kibreab, 2011) showing that ProM is associated with verbal intelligence measured by a variety of tasks including word pronunciation tests [e.g., North American Adult Reading Test (NAART); Blair and Spreen, 1989; Uttl, 2002], word meaning tests (e.g., Short Form Revision of WAIS Vocabulary Test; Jastak and Jastak, 1964), and multiple choice word meaning tests (e.g., Shipley’s Vocabulary test; Shipley, 1940). Recently, Uttl (2011) showed that the association between ProM and verbal intelligence was strong enough to explain why some studies of ProM and aging failed to find age declines in ProM; he found that the verbal intelligence advantage of older adults over younger adults was moderately (*r* = −0.49) correlated with the size of age declines. Notably, the associations between verbal intelligence and vigilance/monitoring in the current study were somewhat stronger than between verbal intelligence and episodic ProM.

## Conclusion

Review of the previous research and the meta-analysis (Study 1) indicates that three of the Big Five factors – conscientiousness, openness, and agreeableness – are weakly related to performance on EC ProM laboratory tasks with correlations ranging from 0.09 to 0.10. However, the meta-analysis was based on a mix of studies that typically did not distinguish between subdomains of ProM, often suffered from a variety of methodological problems, and were generally not powerful enough to detect even a small 0.20 correlation.

Our correlational study with nearly 400 participants (Study 2) revealed no statistically significant associations between any of the Big Five factors and event cued episodic ProM laboratory measures, even though ProM was measured using reliable, ceiling effects free, continuous measures (Graf et al., 2002; Uttl, 2006; Uttl and Kibreab, 2011). However, although we found no statistically significant correlations between episodic ProM and personality in Study 2 (a seemingly inconsistent finding with the meta-analysis of prior studies), the correlations were not statistically different from the estimates derived from the meta-analysis of prior studies. In contrast to episodic ProM, small statistically significant correlations were observed between event cued vigilance/monitoring and two of the Big Five factors – openness and extroversion. Moreover, consistent with a number of prior studies, verbal intelligence was more strongly related to performance on both event cued episodic ProM vigilance/monitoring than measures of personality.

One may find it surprising that we found only a small or no link between at least some personality factors, for example, conscientiousness, and performance on event cued episodic ProM tasks. It is generally believed that conscientious people follow through with their intentions, show up on time, do not miss appointments, don’t forget their promises, are reliable, and are well-organized (Costa and McCrae, 1992; Goldberg et al., 2006; Cuttler and Graf, 2007). Conscientious people are expected to engage in more careful planning of how to fulfill their intentions and consequently succeed in completing them (Cuttler and Graf, 2007). Accordingly, we would expect conscientiousness to correlate positively with EC ProM performance. However, when ProM is assessed in lab settings under tightly controlled experimental conditions, personality factors such as conscientiousness may not have many opportunities to influence task success. In contrast, when ProM is assessed in natural settings, conscientious people may benefit from being able to use their own time-proven strategies to keep their promises and plans such as external reminders (e.g., notes, calendars, smart phones). Consistent with this idea, conscientiousness was correlated with self-reported use of ProM strategies and aids, measured by several different questionnaires, in a large sample of undergraduate students (Uttl and Kibreab, 2011) and conscientiousness was also correlated with a lower frequency of self-reported ProM failures in everyday life (Gondo et al., 2010; Uttl and Kibreab, 2011). Given that external reminder systems are more readily available for time cued vs. event cued tasks, personality factors such as conscientiousness may be the most influential in naturalistic settings and on time cued episodic tasks and the least influential in lab settings and on event cued episodic ProM tasks.

Ours is the first study that examined the relationship between personality and event cued episodic ProM and between personality and vigilance/monitoring within a single study. In contrast to event cued episodic ProM, we found that vigilance/monitoring was associated with openness and extroversion. As noted above, openness and extroversion may help participants to orient themselves in novel laboratory environments, engage with novel tasks, and to figure out strategies for succeeding on vigilance/monitoring tasks, for example, actively keeping the plan in consciousness and looking out for cues. While these strategies are likely to be helpful on vigilance/monitoring, where the time delay between the adoption of a strategy and the appearance of the first cue is short, a few minutes at most, it is not likely to be helpful in keeping a plan in consciousness for longer delays and/or bringing the plan back to consciousness after it has been lost from it.

Thus, our findings highlight that personality factors may affect the various subdomains of ProM differently, leading to at least the following recommendations: First, researchers need to distinguish between the various subdomains of ProM (Graf and Uttl, 2001; Uttl, 2008, 2011) and each study should state clearly which subdomain of ProM was investigated by which ProM task. At present, only a careful reading of the method section allows a reader to determine the sudbomain(s) of ProM investigated in a particular study (Uttl, 2008). Second, researchers should not combine performance on ProM tasks measuring different subdomains into a single composite, for example, performance on a vigilance/monitoring and on an episodic ProM task into a single composite measure of ProM. To illustrate, Salthouse et al. (2004) combined four different ProM tasks, some clearly vigilance/monitoring tasks and some episodic ProM tasks, into a single composite measure and correlated this composite measure with personality factors. However, our findings suggest that how this composite measure correlates with personality factors is merely an artifact of a particular task blend. And third, researchers may need to limit their claims about personality and ProM to only those specific subdomains investigated in their studies. In combination, our findings strongly suggest that the relationship between ProM and personality factors depend on the ProM subdomain as well as study settings.

## Conflict of Interest Statement

The authors declare that the research was conducted in the absence of any commercial or financial relationships that could be construed as a potential conflict of interest.
